# Intrapopulation Genotypic Variation of Foliar Secondary Chemistry during Leaf Senescence and Litter Decomposition in Silver Birch (*Betula pendula*)

**DOI:** 10.3389/fpls.2017.01074

**Published:** 2017-06-26

**Authors:** Ulla Paaso, Sarita Keski-Saari, Markku Keinänen, Heini Karvinen, Tarja Silfver, Matti Rousi, Juha Mikola

**Affiliations:** ^1^Department of Environmental Sciences, University of HelsinkiLahti, Finland; ^2^Department of Environmental and Biological Sciences, University of Eastern FinlandJoensuu, Finland; ^3^Natural Resources Institute Finland (Luke)Helsinki, Finland

**Keywords:** condensed tannins, genotypic variation, heritability, leaf litter decomposition, lignin, phenolic compounds, secondary metabolites, triterpenoids

## Abstract

Abundant secondary metabolites, such as condensed tannins, and their interpopulation genotypic variation can remain through plant leaf senescence and affect litter decomposition. Whether the intrapopulation genotypic variation of a more diverse assortment of secondary metabolites equally persists through leaf senescence and litter decomposition is not well understood. We analyzed concentrations of intracellular phenolics, epicuticular flavonoid aglycones, epicuticular triterpenoids, condensed tannins, and lignin in green leaves, senescent leaves and partly decomposed litter of silver birch, *Betula pendula*. Broad-sense heritability (*H*^2^) and coefficient of genotypic variation (*CV*_G_) were estimated for metabolites in senescent leaves and litter using 19 genotypes selected from a *B. pendula* population in southern Finland. We found that most of the secondary metabolites remained through senescence and decomposition and that their persistence was related to their chemical properties. Intrapopulation *H*^2^ and *CV*_G_ for intracellular phenolics, epicuticular flavonoid aglycones and condensed tannins were high and remarkably, increased from senescent leaves to decomposed litter. The rank of genotypes in metabolite concentrations was persistent through litter decomposition. Lignin was an exception, however, with a diminishing genotypic variation during decomposition, and the concentrations of lignin and condensed tannins had a negative genotypic correlation in the senescent leaves. Our results show that secondary metabolites and their intrapopulation genotypic variation can for the most part remain through leaf senescence and early decomposition, which is a prerequisite for initial litter quality to predict variation in litter decomposition rates. Persistent genotypic variation also opens an avenue for selection to impact litter decomposition in *B. pendula* populations through acting on their green foliage secondary chemistry. The negative genotypic correlations and diminishing heritability of lignin concentrations may, however, counteract this process.

## Introduction

Plants produce an abundance of diverse secondary metabolites such as phenolics and terpenoids. These compounds were thought to be waste products until Fraenkel (1959) recognized that they have an important role in herbivore defense. Since this early discovery, understanding of the role of secondary metabolites in plant ecology has greatly expanded (Theis and Lerdau, 2003). Besides acting as anti-herbivory agents (Haukioja, 2003; Martemyanov et al., 2015), secondary metabolites can defend plants against microbial attack (Dixon, 2001; Pedras et al., 2003), regulate interactions with beneficial microbes such as mycorrhizal fungi (Akiyama et al., 2005) and protect plants against UV radiation (Tegelberg et al., 2001; Keski-Saari et al., 2005). Secondary metabolites can also remain in leaf litter, and by affecting soil microbial activity, litter decomposition, and nutrient cycling have effects on ecosystem functioning (Northup et al., 1998; Hättenschwiler and Vitousek, 2000; Schweitzer et al., 2004; Kotilainen et al., 2009). While these ‘afterlife’ effects are widely recognized, understanding of metabolite dynamics in plant litter is often based on one dominant group of compounds such as condensed tannins (e.g., Schweitzer et al., 2004, 2008b) and studies on the persistence of a wider range of individual compounds (e.g., Gallet and Lebreton, 1995) have remained scarce. Focusing more on the diversity of less abundant metabolites is encouraged by a recent hypothesis that production of low-abundance, low-molecular weight secondary compounds may serve as a mechanism for trees in tropical forests to enforce energy starvation of soil decomposers and conserve the nutrients in litter, where they are accessible by plant-associated mycorrhizal fungi (Hättenschwiler et al., 2011).

The dynamics of secondary metabolites during leaf senescence and litter decomposition can be linked to their chemical structure (Gallet and Lebreton, 1995), but only rarely have such studies been carried out in sufficient detail or scrutinized in terms of the chemical properties of the compounds. Small differences in the chemical structure of the metabolites may strongly affect the rates of chemical processes during decomposition. For example, in the senescent leaves and shed leaf litter, disruption of cellular membranes releases phenolics from vacuoles into cytosol, where they are exposed to the enzymatic activity of polyphenol oxidases (PPOs). Compounds with catechol moiety (*ortho*-diphenol) are preferential substrates for PPOs (Rawel and Rohn, 2010), and are therefore oxidized more rapidly than compounds that lack vicinal hydroxyl groups in their phenolic ring structure. Such differences may determine the fate of compounds during decomposition as was demonstrated for ^14^C-labeled *para*- and *ortho*-hydroxybenzoic acids in taiga forest soils (Sugai and Schimel, 1993). Phenolic compounds vary widely in their structure and some of them, such as flavonoid glycosides and phenolic acids, decrease in concentration already during leaf senescence, while others, particularly polymers (such as lignin and condensed tannins), withstand decomposition (Gallet and Lebreton, 1995). Structural differences may also affect other chemical properties relevant in decomposition, such as the hydrophobicity and toxicity to the decomposing micro-organisms.

Three alternative hypotheses were recently formulated regarding the factors that control litter chemistry during decomposition (Wickings et al., 2012). The chemical convergence hypothesis states that plant litters start resembling each other over the course of decomposition, while the initial litter quality hypothesis proposes that the initial chemistry can be used to simulate the quality throughout the decomposition. The decomposer control hypothesis underlines the importance of distinct decomposer communities influencing the litter chemistry during decomposition. Wickings et al. (2012) found that the chemistry of different litter types diverged rather than converged, but their results also show that the three hypotheses are not mutually exclusive: the decomposer communities have a key role in regulating changes in litter chemistry although the effects depend strongly on the initial litter quality. Recent studies have also found support for the chemical convergence hypothesis (Wallenstein et al., 2013; Parsons et al., 2014), but conclude that this may be an oversimplification.

Large interspecific variation in the composition and quantity of secondary metabolites is known to lead to profound differences in soil organic matter accumulation and nutrient cycling in terrestrial ecosystems (Wardle et al., 1997). Intraspecific genotypic variation in secondary metabolites can be equally substantial as shown for tree species in *Betula* (Keinänen et al., 1999; Laitinen et al., 2000, 2005), *Populus* (Schweitzer et al., 2008b), *Alnus* (Lecerf and Chauvet, 2008), and *Salix* (Heiska et al., 2007). Genetic variation is a prerequisite for natural selection and evolution, but recent evidence suggests that it can also shape local communities and control ecosystem functioning, especially when found in a dominant plant species (Whitham et al., 2008; Genung et al., 2011; Pastor, 2017). Tree genotypes are known to differ in the composition of fungal and insect communities in their canopies (Barbour et al., 2009) and through leaf litter fall, to affect the composition and functioning of soil microbial communities (Schweitzer et al., 2008a; Madritch and Lindroth, 2011). Differences in litter quality within a population can also lead to differences in carbon and nitrogen fluxes (Madritch and Hunter, 2005). In many of these effects, secondary metabolites play a crucial role (Schweitzer et al., 2008a; Barbour et al., 2009; Madritch and Lindroth, 2011), suggesting that these compounds may be particularly helpful in revealing how natural selection, acting on the genetic structure of a dominant plant population, can drive community composition and ecosystem functioning.

In this study, we focus on the fate of foliar secondary metabolites and the persistence of their genotypic variation through leaf senescence and litter decomposition in a *Betula pendula* Roth population. *Betula pendula* is a common, fast-growing deciduous tree in the northern, and eastern Europe (Atkinson, 1992), where it often dominates early boreal forest succession. Due to its ecological and economic importance in the northern areas, the intra- and interpopulation genotypic variation of *B. pendula* traits have been a subject of intensive research. The studies have covered tree growth (Prittinen et al., 2003; Silfver et al., 2009; Mikola et al., 2014) and physiology (Silfver et al., 2008; Possen et al., 2014) as well as herbivore susceptibility (Rousi et al., 1991, 1997; Pusenius et al., 2002; Sinkkonen et al., 2012) and decomposition of leaf litter (Silfver et al., 2007, 2015). Foliar secondary metabolites (Keinänen and Julkunen-Tiitto, 1998; Laitinen et al., 2000), their genotypic variation and role in herbivore and stress responses (Mutikainen et al., 2000; Yamaji et al., 2003) are equally well known for *B. pendula*. For example, the flavonoid aglycones and triterpenoids found on *B. pendula* leaf surface can impair the growth and survival of *Lymantria dispar* larvae (Martemyanov et al., 2015). The ability of secondary metabolites to explain the link between herbivore resistance and litter decomposition rate in *B. pendula* has also been tested, with no obvious role found (Silfver et al., 2015), but the basic knowledge of alterations in the secondary metabolite profiles and their genotypic variation in leaf senescence and litter decomposition is lacking. Our study was designed to fill this gap of knowledge. We hypothesized that secondary metabolites, ranging from the ample condensed tannins and lignin to other phenolics and triterpenoids of lower concentrations (1) remain through leaf senescence, (2) exhibit significant genotypic variation in the senescent *B. pendula* leaves, and (3) remain and preserve their genotypic variation through the early phase of litter decomposition. These hypotheses, if supported, would manifest the persistence of genotypic variation of foliar chemistry through senescence and decomposition: a prerequisite for the initial litter quality to predict and the selection acting on foliar chemistry to affect litter decomposition.

## Materials and Methods

### Field Sites, Plant Material, and Leaf Sampling

The leaf material was collected from the Kuikanniitty experimental site, established in 1999 on an abandoned agricultural field in Punkaharju, southeast Finland (61°47′ N, 29°21′ E). The trees that grow in Kuikanniitty consist of the micropropagated progeny of 30 *B. pendula* trees (Laitinen et al., 2005), selected from a nearby 0.9-ha forest stand (Laitinen et al., 2000). The source stand was naturally regenerated after 1979 logging and the selected mother trees grow in six groups, located 10–60 m apart. The Kuikanniitty site consists of six replicate blocks, each including two trees of each of the 30 genotypes. Nineteen of these genotypes were used in this study (except that green leaf measurements were restricted to eight genotypes after quality assessment of the analyses, where accumulation of polymeric substances had caused inconsistencies in the determination of peak areas), and of the two trees with the same genotype in each block, one was randomly chosen for the study. In 2008, when the leaves were collected, the trees were on average 11 m tall. The thermal growing season (i.e., the period when the mean daily temperature remains above 5°C) started on April 27, ended on October 29 and had a mean temperature of 10.8°C (Finnish Meteorological Institute).

To analyze the green leaf chemistry, a sample of 30 leaves (every second non-damaged leaf from the tip of a south-side branch, growing at the minimum height of 150 cm) was collected from the trees in five replicate blocks (*n* = 5) on June 26. The collected leaves were immediately frozen in liquid nitrogen. For collecting senescent leaves, two south-side branches (at the height of 140–300 cm) of each tree were enclosed in mesh bags before leaf fall (September 8 to 10) in all six blocks (*n* = 6, the number of replicates was increased to fit the number of blocks in the site of decomposition; see below). The mesh bags were collected after leaf fall (October 28 to 30), their contents were pooled within a tree and random subsamples of leaves were taken for laboratory analyses. Remaining leaves were stored in plastic bags in 4°C until November 5, when 10-g (dry mass equivalent) samples were used to establish litter patches on the ground of a clear-cut, *B. pendula*-*Pinus sylvestris* forest site in Loppi, south Finland (60°36′N, 24°24′E). The soil in this site is post-glacial sorted fine sand with a pH of 5.0 and total C and N concentrations of 6 and 0.3%, respectively, in the upper 0–5 cm layer (Mikola et al., 2014). The ground layer vegetation is dominated by a fern *Pteridium aquilinum* (L.) Kuhn, grasses *Calamagrostis arundinacea* (L.) Roth and *Deschampsia flexuosa* (L.) Trin., and dwarf shrubs *Vaccinium myrtillus* L. and *Vaccinium vitis-idea* L. (Mikola et al., 2014). Using a forest site instead of the Kuikanniitty site (which was established on an agricultural field), we ensured that the litter and metabolites were subjected to decomposition in a forest environment, where the decomposers are adapted to tree litter decomposition. The litter patches were allocated to six replicate blocks (following the blocking factor in the Kuikanniitty site) and were covered, but not enclosed, with a 1-mm mesh to prevent disappearance and mixing of leaves. The senescent leaves used in the patches were not dried for dry mass measurements in order to preserve the microbes, such as endophytes (Saikkonen et al., 2003, 2015), growing on the leaves. The litter patches were allowed to decompose in the field until June 24, 2009 (i.e., for 231 days) when 20 partly decomposed leaves were randomly selected from each patch, stored in –76°C and used for secondary metabolite analyses. The mean litter mass loss at this stage of decomposition was 9% (Silfver et al., unpublished data).

### Analyses of Secondary Metabolites

The subsamples of leaves and litter that were used for extracting secondary metabolites were ground in liquid nitrogen and stored in –76°C until analysed. Upon analysis, the samples were dried overnight in a vacuum centrifuge concentrator. Samples of 40 ± 5 mg were then ground using a stainless steel bead in a TissueLyser for 5 min, extracted in 1 ml of 80% methanol for 30 min, centrifuged (13000 rpm, 2 min), and again extracted with 1 ml of 100% for 10 min. The supernatants were dried in a vacuum concentrator at 45°C and stored at 4°C.

Lignin was determined from the precipitants (5 or 10 mg of leaf or litter sample, respectively) using the method described in Brinkmann et al. (2002) and the weight of the obtained biomass pellet was used as an estimate of the lignin content. The dried supernatant was then resuspended in 1.8 ml of 100% methanol and the concentration of condensed tannins (syn. proanthocyanidins) was determined from a 100-μl aliquot of methanol resuspension using the acid butanol assay (Hagerman, 2002). In the assay, 900 μl butanol-HCl (5%) and 10 μl Fe^3+^ -reagent were added and the suspension was incubated at 90°C for 50 min. After being cooled with ice, the absorbance of the suspension was measured at 550 nm using cyanidin chloride (Extrasynthese, Genay, France) as a quantification standard.

For quantifying the concentrations of small-molecular phenolics and triterpenoids, a 500-μl aliquot of the methanol resuspension was dried in a vacuum centrifuge at 45°C and stored at –20°C. After storage, the samples were dissolved in 250 μl 100% methanol and 250 μl distilled water. High-performance liquid chromatography-mass spectrometry (HPLC-MS) was then performed using Thermo Finnigan LC with the flow split into two between a Thermo LTQ MS (Thermo Finnigan, San Jose, CA, United States) with electrospray ionization (ESI) and a Finnigan PDA detector with a subsequent Corona Ultra charged aerosol detector. The column was C-18 Luna with an inner diameter of 2 mm, length of 150 mm and a particle size of 3 μm (Phenomenex, Denmark). The temperature of the tray was set to 18°C and the column to 40°C. The solvents were (A) 0.1% formic acid (Sigma–Aldrich, Steinheim, Germany) in H_2_O and (B) 0.1 % formic acid in acetonitrile (Chromasolv^®^ grade, Sigma–Aldrich). The flow was 0.41 ml min^-1^ and the elution was performed with a gradient as follows: B started with 5%, was increased to 50% by 15 min, to 60% by 35 min, to 85% by 45 min and to 98% by 50 min and then kept at 98% for 5 min. The column was returned to its starting condition with 15 min equilibration, giving a total of 70 min for each run. The injection volume was 12 μl with a partial loop. The MS was run in a positive ion mode with a mass range of 150–1500 m/z. The capillary temperature was kept at 320°C and the voltage at 5 V. The sheath gas flow rate was kept at 20 ml min^-1^, auxiliary gas flow rate at 5 ml min^-1^ and sweep gas flow rate at 5 ml min^-1^. The tube lens was set to 80 V. The areas for the compounds were integrated using the Xcalibur software.

The compounds were annotated using retention times, UV spectra and HPLC-MS. Peak picking was done using MetAlign software (Lommen, 2009) based on the green leaf samples. All analyzed compounds with their retention times and quantification ions are listed in Supplementary Table S1. The compounds were coded in the order of retention time, which reflects increasing lipophilicity in the reversed phase LC. The quantification was carried out using commercial standards: i.e., chlorogenic acid (Aldrich) for caffeoylquinic acids and their derivatives (CQAs), coumaroylquinic acids (CouQAs) and 3,4′-dihydroxypropiophenone-3-β-D-glucopyranoside (DHPPG); (+)-catechin (Aldrich) for (+)-catechin; quercetin 3-glucoside (Extrasynthese) for myricetin, quercetin, and kaempferol derivatives; and acacetin (Extrasynthese) for flavonoid aglycones. Triterpenoids are reported as arbitrary units (peak area g^-1^ dry mass). For the analyses of heritability and statistical significance of genotypic variation in the senescent leaves and decomposed litter, the small-molecular phenolics and triterpenoids were grouped into intracellular phenolics (including CQAs, CouQAs, DHPPG, (+)-catechin, myricetin glycosides, quercetin glycosides, and kaempferol glycosides), epicuticular flavonoid aglycones and epicuticular triterpenoids. In addition, the intracellular phenolics were tested as subgroups; i.e., phenolic acids (CQAs and CouQAs), myricetin glycosides, quercetin glycosides, and kaempferol glycosides.

### Statistical Analyses

To avoid a multitude of mean tests and to allow easy statistical inference in the graphs (Cumming, 2009), we interpreted the statistical significance of differences between green leaf, senescent leaf and decomposing litter metabolite concentrations using 85% confidence intervals (CIs) of means. In this approach, non-crossing CIs of two means denote a statistically significant difference between the means. It is a common practice to use 95% CIs, but they are too conservative for testing mean differences and the best approximation of α = 0.05 is achieved using 85% CIs (Payton et al., 2000). All concentration means were calculated using data from genotypes 5, 6, 8, 12, 14, 15, 20, and 25 as these were available for green leaves.

Since our plant material consisted of a cloned progeny of the selected *B. pendula* genotypes, all trees within a genotype had an equal genetic structure. In such material, all variation that is found within genotypes can be considered to be due to the variation in environment, or due to a measurement error, and all variation found between the genotypes to be genetic (Falconer, 1989). This genotypic variation includes both the additive and non-additive components, which cannot be separated in cloned material, and only the degree of genetic determination, i.e., the broad-sense heritability (*H*^2^) can be calculated (Falconer, 1989). In our study, the broad-sense heritabilities of the concentrations of the three metabolite groups (intracellular phenolics, epicuticular flavonoid aglycones, and epicuticular triterpenoids), intracellular phenolic subgroups, soluble condensed tannins and lignin were calculated on individual plant basis according to the Eq. 1, where $\sigma_{G}^{2}$ and $\sigma_{E}^{2}$ are variance components for genotypes and error, respectively (calculated using the SPSS GLM Variance components procedure). Following common practice in forest breeding, the replicate block was included in the calculation model as a fixed factor (which removes the block-scale variation from error variance). This differs from our earlier *B. pendula* studies in a natural forest site (Mikola et al., 2014; Silfver et al., 2015), where we were also interested in the size of the block-scale environmental variation and treated block as a random factor.

(1) $$H^{2}=\sigma_{G}^{2}/\left(\sigma_{G}^{2}+\sigma_{E}^{2}\right)$$

Coefficients of genotypic variation (*CV*_G_) were calculated according to the Eq. 2, where 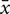 is the phenotypic mean.

(2) $${CV}_{G}=\sqrt{\sigma_{G}^{2}/\overset{-}{X}}$$

The statistical significance of genotypic variation in the concentrations of the three groups of small-molecular metabolites, intracellular phenolic subgroups, condensed tannins, and lignin was tested using the analysis of variance. Following the heritability calculations, the genotype was treated as a random factor and the field replicate block as a fixed factor. The homogeneity of residual variance among genotypes was tested using the Levene’s test and the normal distribution of model residuals using the Shapiro–Wilk test. To fulfill the variance and normality assumptions, the data of condensed tannins was log_10_ transformed and the data of other metabolites, excluding lignin, square-root transformed. Lignin data fulfilled the assumptions without a transformation.

Relations among genotypes and individual secondary metabolites were further examined using the principal component analysis (PCA). Compounds with qualitative variation were excluded from these analyses and the senescent leaves and decomposed litter were analyzed separately as not all the compounds of senescent leaves were present in litter. Before PCA, both columns and rows of the data matrix were transformed to have a mean of zero and a standard deviation of 1. This was done to reduce the quantitative differences among the compounds and the samples and thus, focus more on chemical profiles. Compounds with skewed distribution were log_10_ transformed. The significance of genotypic variation along the first two PC axes was analyzed using axis scores and the same ANOVA models as used for compound concentrations.

Genotypic correlations among the compound groups and between the senescent leaves and decomposed litter were tested using the Spearman rank correlation analysis. The persistence of genotypic variation in the chemical profiles revealed by PCA between the senescent leaves and litter was tested as rank correlations of the genotype means of PC axis scores. All statistical analyses were performed using the SPSS 15.0.1 and SPSS 18 statistical packages (SPSS, Chicago, IL, United States) except for the PCA, which was performed using the SIMCA-P+ software (Umetrics AB, Umeå, Sweden).

## Results

### Changes in Metabolite Concentrations during Leaf Senescence and Litter Decomposition

**FIGURE 1:**
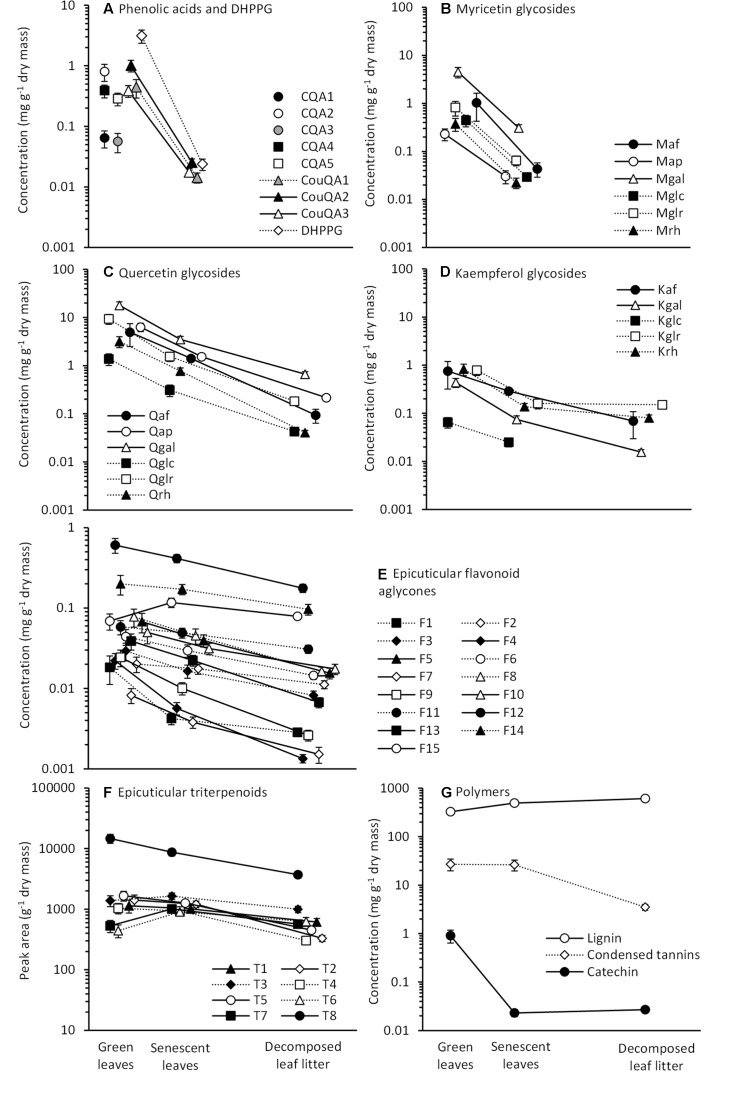
Mean concentrations (±85% CI, *n* = 5 for arabinofuranosides and 33–38 for other compounds, based on genotypes 5, 6, 8, 12, 14, 15, 20, and 25) of phenolic compound groups: **(A)** phenolic acids and DHPPG, **(B)** myricetin glycosides, **(C)** quercetin glycosides, **(D)** kaempferol glycosides, **(E)** epicuticular flavonoid aglycones, **(F)** epicuticular triterpenoids and **(G)** the polymers in *Betula pendula* green leaves, senescent leaves and decomposed leaf litter (the interval between green and senescent leaves is 4 months and between senescent leaves and decomposed litter 8 months; triterpenoids are reported as peak area; CQA, Caffeoylquinic acid; CouQA, Coumaroylquinic acid; DHPPG, 3,4′-dihydroxypropiophenone-3-glucoside; M, Myricetin; Q, Quercetin; K, Kaempferol; af, arabinofuranoside; ap, arabinopyranoside; gal, galactoside; glc, glucoside; glr, glucuronide; rh, rhamnoside).

All those secondary metabolites that were found in green summer leaves were also detected in senescent leaves, except for CQAs (**Figure 1A**). The concentrations of CouQAs were on average 97% lower in the senescent than green leaves and decreased below the detection limit during litter decomposition (**Figure 1A**). DHPPG concentration was 99% lower in the senescent than green leaves and also decreased below detection during decomposition (**Figure 1A**).

The decrease of flavonol glycoside concentrations during leaf senescence and litter decomposition varied among the flavonoid subgroups (**Figures 1B–D**). Concentrations of myricetin glycosides decreased on average by 93% during leaf senescence and none of the six compounds was detected in the decomposed litter (**Figure 1B**). In contrast, concentrations of quercetin and kaempferol glycosides decreased on average by 79 and 76% during leaf senescence and all compounds, except for kaempferol-3-glucoside, were also detected in the decomposed litter (**Figures 1C,D**). Based on the comparison of confidence intervals, the reduction in concentration during leaf senescence was statistically significant for all flavonol glycosides, except for kaempferol 3-arabinofuranoside (**Figures 1B–D**). During litter decomposition, the concentration of quercetin and kaempferol glycosides decreased on average by 86 and 52%, and except for kaempferol 3-glucuronide, the decrease was statistically significant in all compounds (**Figures 1C,D**).

The concentrations of epicuticular flavonoid aglycones varied a lot in the green leaves, but displayed relatively similar dynamics during leaf senescence and litter decomposition (**Figure 1E**). The concentrations were on average 27% lower in the senescent than green leaves and the decrease was statistically significant for nine of the 15 compounds (**Figure 1E**). For one of the compounds (F15), the concentration increased by 70% (**Figure 1E**). During litter decomposition, the concentrations of flavonoid aglycones decreased on average by 51% and the decrease was statistically significant for all compounds (**Figure 1E**).

The mean concentration of triterpenoids decreased during leaf senescence by 25% (**Figure 1F**), but this decrease was driven by one abundant compound T8 that was annotated as 12-*O*-acetyl-3-O-malonylbetulafolientriol. When T8 was excluded from calculations, the mean concentration of triterpenoids increased by 4%, and for the two ocotillol-type triterpenoids, papyriferic acid (T7) and its derivative (T6), the increase was statistically significant (**Figure 1F**). During litter decomposition, all triterpenoids had parallel dynamics, the mean concentration decreased by 55% and the decrease was statistically significant for all compounds (**Figure 1F**).

Of the polymers, the concentration of lignin increased by 51%, while the concentration of condensed tannins did not change during leaf senescence (**Figure 1G**). During litter decomposition, lignin concentration increased further by 23%, but tannin concentration decreased by 87% (**Figure 1G**). The (+)-catechin concentration decreased by 97% during senescence, but did not change during decomposition (**Figure 1G**).

### Genotypic Variation in Metabolite Concentrations

The secondary metabolites displayed both qualitative (absence or presence in only certain genotypes) and quantitative (found in all genotypes, but in varying quantity) genotypic variation. Qualitative variation was found among flavonol glycosides: the 3-glucuronides were lacking in four of the 19 genotypes (16, 24, 25 and 30) and the 3-arabinofuranosides were found in four genotypes only (2, 8, 22, and 23). The qualitative variation remained through the senescence and decomposition as these compounds were also found in the decomposed litter (**Figures 1B–D**).

**FIGURE 2:**
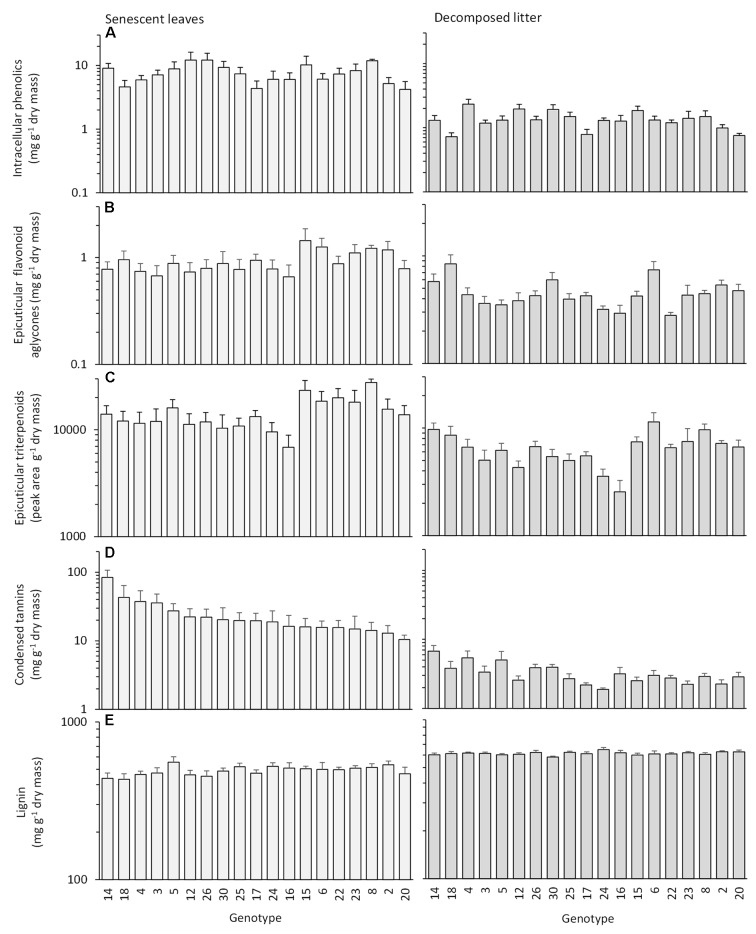
Concentrations of **(A)** intracellular phenolics, **(B)** epicuticular flavonoid aglycones, **(C)** epicuticular triterpenoids, **(D)** condensed tannins, and **(E)** lignin (mean + SE, *n* = 5–6) in the senescent leaves and decomposed litter of 19 *Betula pendula* genotypes (arranged in a decreasing order of senescent leaf tannin concentrations).

Quantitative genotypic variation was found in all compound groups (**Figure 2** and **Table 1**). Of the main metabolite groups, the epicuticular triterpenoids (**Figure 2C**) had the highest broad-sense heritability, *H*^2^ (0.281) and coefficient of genotypic variation, *CV*_G_ (0.138) in the senescent leaves (**Table 1**). The other groups had very similar heritabilities (0.113–0.121), whereas the *CV*_G_ varied more, with lignin and epicuticular flavonoid aglycones having lower *CV*_G_ than intracellular phenolics and condensed tannins (**Table 1**). The genotypic variation was statistically not highly significant in the senescent leaves, except for triterpenoids (**Table 1**). Among the intracellular phenolic subgroups, myricetin glycosides and kaempferol glycosides had very high values of *H*^2^ (0.398 and 0.327, respectively) and *CV*_G_ (0.331 and 0.219), while those of phenolic acids and quercetin glycosides resembled the values of intracellular phenolics in general (**Table 1** and Supplementary Figure S1). During decomposition, the *H*^2^ and *CV*_G_ increased for intracellular phenolics, epicuticular flavonoid aglycones, and condensed tannins, remained the same for epicuticular triterpenoids and decreased for lignin (**Figure 2** and **Table 1**). As a result, the decomposed litter had statistically highly significant genotypic variation in all compounds except for lignin that had lost genotypic variation during decomposition (**Table 1**).

**Table 1:** The variance components (σ^2^), broad-sense heritability (*H*^2^), phenotypic mean (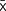), coefficient of variation (*CV*_G_), and *F* and *P* statistics of ANOVA of the genotypic variation of secondary metabolites in the senescent leaves and decomposed litter of *Betula pendula* (G = genotype, E = error; means are mg g^-1^ dry mass, except for epicuticular triterpenoids peak area g^-1^ dry mass; lignin not transformed, condensed tannins log_10_-transformed, other groups square root-transformed; bold values denote statistically significant genotype effects).

	σ_G_^2^	σ_E_^2^	*H*^2^	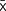	*CV*_G_	*F*	*P*
**Senescent leaves**							
Intracellular phenolics	0.080	0.617	0.115	2.58	0.110	1.69	0.060
Phenolic acids	4.7E-4	0.003	0.120	0.22	0.098	1.61	0.086
Myricetin glycosides	0.037	0.056	0.398	0.58	0.331	4.76	**<0.001**
Quercetin glycosides	0.077	0.539	0.125	2.43	0.114	1.76	**0.047**
Kaempferol glycosides	0.015	0.031	0.327	0.56	0.219	3.58	**<0.001**
Epicuticular flavonoid aglycones	0.005	0.038	0.116	0.92	0.077	1.67	0.062
Epicuticular triterpenoids	247	632	0.281	114	0.138	3.08	**<0.001**
Condensed tannins	0.018	0.142	0.113	1.19	0.113	1.95	**0.021**
Lignin	496	3613	0.121	491	0.045	1.79	**0.040**
**Decomposed litter**							
Intracellular phenolics	0.021	0.057	0.269	1.13	0.128	2.99	**<0.001**
Quercetin glycosides	0.019	0.058	0.248	1.00	0.139	2.76	**0.001**
Kaempferol glycosides	0.006	0.005	0.528	0.49	0.158	6.96	**<0.001**
Epicuticular flavonoid aglycones	0.005	0.020	0.200	0.66	0.107	2.40	**0.004**
Epicuticular triterpenoids	115	352	0.246	79	0.136	2.75	**0.001**
Condensed tannins	0.011	0.032	0.263	0.47	0.223	3.01	**<0.001**
Lignin	29	874	0.032	614	0.009	1.19	0.289

**FIGURE 3:**
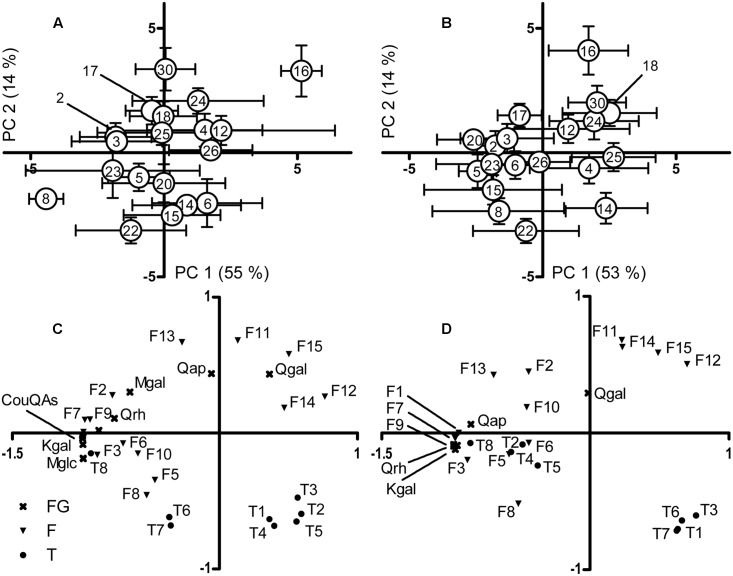
Principal component analysis (PCA) graphs of the secondary metabolite data in **(A)** senescent leaves and **(B)** decomposed litter and the loadings of the compounds responsible for the variation on the PC axes as *p*(corr) values in **(C)** senescent leaves and **(D)** decomposed litter. In **(A,B)**, the 19 genotypes are shown as mean axes scores with the vertical and horizontal error bars depicting ±1 SE (*n* = 4–6). CouQAs, Coumaroylquinic acids; F, Flavonoid aglycone; FG, Flavonol glycosides; Kgal, Kaempferol 3-galactoside; Krh, Kaempferol 3-rhamnoside; Mgal, Myricetin 3-galactoside; Mglc, Myricetin 3-glucoside; Qap, Quercetin 3-arabinopyranoside; Qgal, Quercetin 3-galactoside; Qrh, Quercetin 3-rhamnoside; T, Triterpenoid.

The genotypic variation in the small-molecular compounds of senescent leaves was also clearly visible in the PCA of individual compounds, where PC1 represents environmental variation (*P* = 0.172 for genotype, *P* < 0.001 for replicate block) and PC2 mostly genotypic variation (*P* < 0.001 for genotype, *P* = 0.042 for block) (**Figure 3A**). In the decomposed litter, genotypic variation was significant along both the PC1 (*P* = 0.018 for genotype, *P* < 0.001 for block) and the PC2 (*P* < 0.001 for genotype, *P* = 0.003 for block) (**Figure 3B**). The ranks of genotype mean scores correlated positively between the senescent leaves and decomposed litter for PC2 (ρ = 0.87, *P* < 0.001), but not for PC1 (ρ = –0.43, *P* = 0.064). The genotype 16 was most distinct from others in both senescent leaves and decomposed litter (**Figures 3A,B**). Triterpenoids and the most lipophilic flavonoid aglycones (F11-F15) were the compounds that best explained the genotypic variation along the PC axes (**Figures 3C,D**). In the decomposed litter, four triterpenoids, including papyriferic acid (T7) and its derivative (T6), formed a tight cluster separated from the rest of the compounds (**Figure 3D**).

### Genotypic Correlations among Metabolites

The genotype mean concentrations of the two epicuticular compound groups – flavonoid aglycones and triterpenoids – were positively correlated in the senescent leaves, whereas the mean concentrations of condensed tannins correlated negatively with the concentrations of flavonoid aglycones and lignin (**Table 2** and Supplementary Figure S2). The positive correlation between the flavonoid aglycones and triterpenoids also remained in the decomposed litter (**Table 2**). Among the intracellular phenolic subgroups of the senescent leaves, concentrations of phenolic acids correlated positively with the concentrations of quercetin glycosides (ρ = 0.63, *P* = 0.004, *n* = 19) and kaempferol glycosides (ρ = 0.52, *P* = 0.023), which also correlated with each other (ρ = 0.63, *P* = 0.004). The ranks of genotype mean concentrations correlated positively between the senescent leaves and decomposed litter for intracellular phenolics (ρ = 0.70, *P* = 0.001, *n* = 19), flavonoid aglycones (ρ = 0.46, *P* = 0.048), triterpenoids (ρ = 0.75, *P* < 0.001) and condensed tannins (ρ = 0.62, *P* = 0.004), but not for lignin (ρ = 0.16, *P* = 0.514) (**Figure 2**).

**Table 2:** Rank correlation coefficients (Spearman’s rho) of genotype mean concentrations of secondary metabolites in the senescent leaves and decomposed litter of *Betula pendula* (*n* = 19; ^∗^*P* < 0.05, ^∗∗^*P* < 0.01, ^∗∗∗^*P* < 0.001; bold values denote statistically significant correlations).

	Epicuticular flavonoid aglycones	Epicuticular triterpenoids	Condensed tannins	Lignin

**Senescent leaves**				
Intracellular phenolics	0.05	0.14	0.17	<0.01
Epicuticular flavonoid aglycones		**0.75^∗∗∗^**	-**0.47^∗^**	0.25
Epicuticular triterpenoids			-0.43	0.15
Condensed tannins				-**0.51^∗^**
**Decomposed litter**				
Intracellular phenolics	-0.02	-0.05	0.15	-0.37
Epicuticular flavonoid aglycones		**0.71^∗∗^**	0.29	-0.13
Epicuticular triterpenoids			0.20	-0.25
Condensed tannins				-0.37

## Discussion

Our results show that although the concentrations of many secondary metabolites decreased significantly during *B. pendula* leaf senescence, all metabolites except for caffeoylquinic acids (CQAs) remained in the senescent leaves. As we hypothesized, the remaining metabolites also exhibited significant genotypic variation with high broad-sense heritabilities and coefficients of genotypic variation. During decomposition, most metabolites decreased in concentration, suggesting that they were decomposed faster than the litter material on average, but the genotypic variation was persistent. This was manifested by the increasing heritabilities and coefficients of genotypic variation of the intracellular phenolics, surface flavonoid aglycones and condensed tannins during litter decomposition. Confirming the persistence of genotypic variation, the genotype ranks in metabolite concentrations remained stable under field conditions and microbial degradation. Considering that secondary metabolites can affect litter decomposition and nutrient cycling (Hättenschwiler and Vitousek, 2000; Schweitzer et al., 2004), these results suggest that by acting on the foliar secondary metabolite profiles of *B. pendula* populations, selection can be a significant driver of litter decomposition. Lignin was an important exception among the compounds, however, as lignin concentrations increased during leaf senescence and litter decomposition and the genotypic variation disappeared during decomposition. Moreover, our results show that the genotype means of secondary metabolite concentrations, like those of condensed tannins and lignin, can be negatively correlated in the senescent leaves. As lignin (Melillo et al., 1982; Hobbie et al., 2006; Talbot and Treseder, 2012) and condensed tannins (Schweitzer et al., 2004, 2008a) can both restrict litter decomposition, an association between litter decomposition rate and a concentration gradient of one compound could be canceled out by an inverse gradient of the other. In such case, selection acting on the concentration of either compound in the green leaves might not lead to a significant change in litter decomposition rate in the population. Also, equally important is to recognize the role of other characteristics of litter chemistry, such as concentrations of nitrogen (Silfver et al., 2007) and micronutrients (Makkonen et al., 2012; García-Palacios et al., 2016a), when weighing the opportunity of selection to drive decomposition through changes in green leaf secondary chemistry.

### Metabolite Dynamics during Senescence and Decomposition

The absence of CQAs in the senescent leaves was expected due to their catechol (*o*-diphenol) moiety, which makes them preferential substrates for PPOs (Rawel and Rohn, 2010). Monophenols, such as CouQAs, are oxidized less efficiently, either by PPOs or through non-enzymatic reactions, which most likely explains their slower degradation. The persistence of flavonol glycosides also appeared to depend on the number of adjacent hydroxyl groups in their chemical structure: the concentration of myricetin glycosides, which have three adjacent hydroxyl groups in their B-ring, decreased on average by 93% during senescence and disappeared during decomposition, whereas the quercetin and kaempferol glycosides, which have two and one hydroxyl groups, respectively, were more persistent. Non-enzymatic oxidation also likely contributed to decreasing flavonol glycoside concentrations and may partly explain differences in their degradation rates. In green leaves, the flavonol glycosides reside in slightly acidic vacuoles, but organelle disruption in leaf senescence exposes them to higher pH. The oxidation rate of flavonols is markedly elevated at higher pH (7.5), but while myricetin oxidizes substantially faster than quercetin, in the same conditions kaempferol does not oxidize at all (Canada et al., 1990).

The flavonoid aglycones, which situate on leaf surface (Keinänen and Julkunen-Tiitto, 1998), had a low concentration (under 0.1 mg g^-1^ dry mass) in the green leaves, but lost little of their concentration during leaf senescence. Epicuticular flavonoids are typically highly methylated, which makes them lipophilic, but at the same time shields the reactive hydroxyl groups from oxidation (Rice-Evans et al., 1996). During litter decomposition, the flavonoid aglycones lost around a half of their concentration, suggesting that their degradation rate was significantly higher than the average decomposition rate of the litter material. In the PCA, however, the most lipophilic (highly methylated) flavonoid aglycones clustered separately from the more hydrophilic ones, which likely indicates their higher oxidative stability. Similarly, to our results, methylated flavonoid aglycones (kaempferol and apigenin derivatives) were shown to be persistent in the soils underneath the shrub *Cistus ladanifer*, with low concentrations remaining for more than 16 months without further input (Sosa et al., 2010). The other group of epicuticular compounds, the triterpenoids, was also among the most persistent compounds during leaf senescence, with only a small decrease or even an increase in concentration (except for the most abundant compound, 12-*O*-acetyl-3-*O*-malonylbetulafolientriol). The two triterpenoids that increased in concentration during senescence were both ocotillol-type of oxidized dammaranes (papyriferic acid, T7 and its derivative, T6). These are known to be formed from corresponding dammaranes in oxidative conditions, e.g., through UV-induced photochemical reactions (van der Doelen and Boon, 2000). It is thus possible that the main triterpenoid, 12-*O*-acetyl-3-*O*-malonylbetulafolientriol was partly oxidized to the corresponding oxide, papyriferic acid (12-*O*-acetyl-3-*O*-malonylbetulafolientriol oxide I) during the senescence. In the PCA of decomposed litter, two other triterpenoids (T1 and T3) clustered together with the oxidized ocotillol-type triterpenoids (T6 and T7), which suggests that the concentration pattern of these compounds was also influenced by oxidative processes.

As could be expected, the polymers were among the most persistent metabolites during leaf senescence and decomposition. Consistent with an earlier study with mountain birch *B. pubescens* ssp. *czerepanovii* (Stark et al., 2007), we found that the concentration of condensed tannins did not change during leaf senescence. Instead, the concentration dropped significantly during early decomposition. Considering that condensed tannins can slow the rate of litter decomposition (Schweitzer et al., 2004, 2008b) this seems unexpected, but a similar drop has earlier been found, e.g., in the litter of *Betula papyrifera* (Parsons et al., 2004) and may partly be explained by leaching (Schofield et al., 1998). The concentration of (+)-catechin, a common monomeric building unit of condensed tannins, decreased during leaf senescence, but not during litter decomposition. This implies that condensed tannins broke down into the (+)-catechin units during decomposition (Jorgensen et al., 2004). Contrary to other metabolites, lignin concentration increased during leaf senescence and litter decomposition, the pattern being very close to that found by Berg and Ekbohm (1991) for *B. pendula*. The increasing concentration was expected as lignin is recalcitrant to decomposition and other major constituents such as carbohydrates and cellulose are degraded faster than lignin (Zimmer, 2002; Preston et al., 2009).

### Genotypic Variation of Secondary Metabolites

While there has long been evidence of intraspecific genetic variation in the composition and concentrations of secondary metabolites in leaf litter (e.g., Schweitzer et al., 2008b), the persistence of this variation during litter decomposition has remained unexplored. Here, we show that the genetic variation in foliar chemistry can remain high and even increase under the decomposer activity and abiotic conditions of the forest floor. The coefficient of genotypic variation (*CV*_G_) of growth has earlier been estimated to vary between 0.10 and 0.19 for those trees, from which we collected the litter (Mikola et al., 2014), and between 0.05 and 0.13 for other *B. pendula* populations (Stener and Hedenberg, 2003; Stener and Jansson, 2005). Our *CV*_G_ estimates for the metabolite concentrations of the senescent *B. pendula* leaves and decomposing litter are in the same range, and remarkably, the *CV*_G_ of tannin concentrations in the decomposing leaf litter surpasses these estimates of living trees.

When interpreting broad-sense heritabilities, one needs to be careful as *H*^2^ values depend on both the genotypic and environmental variation and decreasing environmental variation alone can lead to increasing heritability values. However, unlike the *H*^2^ estimates, the *CV*_G_ estimates do not depend on the environmental variation and are thus a more reliable estimate of the genotypic variation (Houle, 1992; Hansen et al., 2011). In our study, the environmental variation was higher in the metabolites of senescent leaves collected from trees grown at Kuikanniitty than in the metabolites of litter decomposed at the Loppi site. Such difference is likely related to dissimilarities in environmental variation between the sites, and to the ability of field blocking to explain and remove this variation, and could alone explain the increasing *H*^2^ estimates that we found during decomposition. However, as the increasing heritabilities were accompanied by increasing *CV*_G_ values in many metabolites, the increasing genotypic variation during decomposition cannot be an artifact caused by decreasing environmental variation. Moreover, confirming the persistence of genotypic variation, we found that the genotype ranks in metabolite concentrations remained stable. Our results can thus be interpreted to illustrate a remarkable after-life genotypic variation of foliar chemistry in local *B. pendula* populations. Through this variation, selection acting on foliar chemistry should have a potential to affect decomposition and nutrient dynamics. An example of such selection in action is provided by Bryant et al. (2009), who showed that across the North American boreal forest zone, areas with high incidence of forest fires support high population densities of hares, which in turn selects for genotypes of Alaska birch (*Betula neoalaskana* Sargent) that have high papyriferic acid production. Recent results also suggest that the concentrations of condensed tannins in leaf litter could be the target of selection due to the beneficial effects of high tannin concentrations on plant nitrogen uptake in certain conditions (Madritch and Lindroth, 2015).

The PCA revealed that the genotypic variation was compound-specific and greatly affected by the epicuticular compounds, i.e., flavonoid aglycones and triterpenoids. To our knowledge, the fate of these compounds has not earlier been examined in leaf litter, and while their concentrations were mostly low, their role in genotypic variation was substantial. For instance, the papyriferic acid (T7), which is known to defend birch twigs against hare browsing (Reichardt et al., 1984; Rousi et al., 1991, 1996), was one of the major components of genotypic variation. Intracellular flavonol glycosides, in contrast, exhibited mainly variation among replicate blocks. This agrees with a recent study, in which the phenolic profile of leaf litter exhibited environmental rather than genotypic variation (Zimmer et al., 2015). Even though the genotypic variation of most secondary metabolites remained high in our study, the loss of genotypic variation in lignin concentrations implies that genotypic variation in the ‘general litter quality’ may be diminished during decomposition. Indeed, our study suggests that litter decomposition of *B. pendula* genotypes has characteristics from both the chemical convergence (considering lignin) and initial litter quality hypothesis (most other metabolites) (cf. Wickings et al., 2012). The initial differences in litter quality were recently found to play a major role in the later stages of litter decomposition (García-Palacios et al., 2016b). Our results suggest that one potential reason for this could be that some of the initial differences in litter chemistry get stronger during decomposition. The strong genotypic variation in litter chemistry in our study implies that differences in the composition and quantity of phenolic compounds among the trees within a local population may have community-wide effects. Leaf litter phenolics can affect mycorrhizal fungal communities (Piotrowski et al., 2008) and inhibit plant seedling and root growth (Bonanomi et al., 2011), and since phenolic compounds are commonly activated by oxidative conditions and soils harbor abundant microbial enzymes and oxidants, soils have high potential for phenolic activity (Appel, 1993).

### Genotypic Correlations

Except for lignin, the genotype means of metabolite concentrations, as well as the axis scores of PC2 that depicted the genotypic variation of metabolite profiles, were positively correlated between the senescent leaves and decomposing litter. This shows that the genotypic variation remains through the early decomposition (9% of litter mass was lost before sampling; Silfver et al., unpublished data), which is a prerequisite for a long-term genetic influence on decomposition. However, we also found significant genotypic correlations among the compound groups. Of these, the positive correlation between flavonoid aglycones and triterpenoids has earlier been found in the green leaves (Lihavainen et al., 2017) and bark (Laitinen et al., 2004) of *B. pendula*. Epicuticular flavonoid aglycones and triterpenoids are synthesized in multicellular peltate glands on birch leaves and twigs and excreted to the surface as a mixed resin to provide chemical defense and protect from desiccation (Raatikainen et al., 1992; Valkama et al., 2003). By contrast, the flavonoid aglycones correlated negatively with vacuolar condensed tannins that consist of monomeric flavonoid units. Because of their localization in different parts of the leaf lamina, the negative correlation may not indicate a biosynthetic trade-off, which is further supported by our finding that intracellular phenolics, which comprised mainly of flavonol glycosides, did not show a negative correlation with condensed tannins. Instead, condensed tannins correlated negatively with the other major phenolic polymer, lignin. This association has rarely been reported, but may have significant consequences on the effects of selection on litter decomposition. We are aware of only one earlier study with *Populus tremuloides*, where Madritch and Lindroth (2011) showed that concentrations of lignin and condensed tannins in leaf litter exhibited strong genotypic variation and that high tannin genotypes displayed low lignin content and *vice versa*.

## Conclusion

The green foliage secondary chemistry of a tree population is a reflection of various selection forces, such as herbivory, which act on the genotypic structure of the population. Our results show that a majority of those secondary metabolites that have earlier been found to characterize *B. pendula* green foliage remain in the senescent leaves, although in much lower concentrations, and can withstand the first phase of decomposition. The persistence of the compounds appears to be related to their chemical properties, and particularly to their susceptibility to oxidation. It further appears that the genotypic variation of metabolite concentrations persists through leaf senescence and litter decomposition. This supports the hypothesis that initial litter quality can explain the variation in litter decomposition rates. It also opens an avenue for selection to impact litter decomposition in *B. pendula* populations through acting on their green foliage secondary chemistry. Whether this takes place, however, likely depends on the relative strength of the link of litter decomposition to secondary metabolites in comparison to the other attributes of *B. pendula* litter. At a particular forest site, finally, the significance of changes in litter metabolite concentrations caused by the selection apparently depends on the relative importance of litter chemistry among other biotic and environmental factors that control litter decomposition rates.

## Author Contributions

JM designed the study and MR arranged the plant material; UP and HK carried out the field and laboratory work; TS contributed to the field work and SK-S and MK guided the laboratory analyses; UP, SK-S, and MK interpreted the data; JM, UP, SK-S, and TS analyzed the data; UP and JM wrote the manuscript and SK-S, MK, MR, and TS developed the text.

## Conflict of Interest Statement

The authors declare that the research was conducted in the absence of any commercial or financial relationships that could be construed as a potential conflict of interest.
