# Supplementary material for: Intrapopulation Genotypic Variation of Foliar Secondary Chemistry during Leaf Senescence and Litter Decomposition in Silver Birch (Betula pendula)
Source: Front Plant Sci. 2017 Jun 26;8:1074. doi: 10.3389/fpls.2017.01074 (PMC5483462; doi:10.3389/fpls.2017.01074)
Supplement: Supplementary file 1 [file Data_Sheet_1.docx]

Supplementary Material

Intrapopulation genotypic variation of foliar secondary chemistry during leaf senescence and litter decomposition in silver birch (*Betula pendula*)

**Ulla Paaso, Sarita Keski-Saari, Markku Keinänen, Heini Karvinen, Tarja Silfver, Matti Rousi, Juha Mikola***

***Correspondence** Juha Mikola: [juha.mikola@helsinki.fi](mailto:juha.mikola@helsinki.fi)

# Supplementary Tables and figures

## Tables

| **Supplementary Table 1.** The analyzed secondary metabolites, their abbreviation, retention time (RT), quantification ion, base peak (m/z+1) and other ions. Within flavonoid aglycones and triterpenoids, the metabolites are named using ordinal numbers in the order of retention time. | | | | | |
| --- | --- | --- | --- | --- | --- |
| Compound | Abbr. | RT | Ion(s) | Base peak | Other ions |
|  |  |  |  |  |  |
| **Intracellular phenolics** |  |  |  |  |  |
| 5-Caffeoylquinic acid | CQA1 | 6.1 | 355 | 355 |  |
| 3,4'-Dihydroxypropiophenone-3-glucoside | DHPPG | 8 | 329 | 329 |  |
| Coumaroylquinic acid 1 | CouQA1 | 8.6 | 339 | 339 |  |
| (+)-Catechin | Catechin | 8.9 | 291 | 291 |  |
| 3-Caffeoylquinic acid (chlorogenic acid) | CQA2 | 9.4 | 355 | 355 |  |
| 4-Caffeoylquinic acid | CQA3 | 11.7 | 355 | 355 |  |
| Coumaroylquinic acid 2 | CouQA2 | 12.2 | 339 | 339 |  |
| Coumaroylquinic acid 3 | CouQA3 | 14.4 | 339 | 339 |  |
| Caffeoylquinic acid derivative 4 | CQA4 | 15.6 | 355 | 355 |  |
| Caffeoylquinic acid derivative 5 | CQA5 | 16.8 | 355 | 355 |  |
| *Flavonol glycosides* |  |  |  |  |  |
| Myricetin 3-galactoside | Mgal | 14.7 | 481 | 481 | 319 |
| Myricetin 3-glucoside | Mglc | 15.2 | 481 | 481 | 319 |
| Myricetin 3-glucuronide | Mglr | 15.7 | 495 | 495 | 319 |
| Myricetin 3-arabinopyranoside | Marap | 16.4 | 451 | 451 | 319 |
| Myricetin 3-arabinofuranoside | Maraf | 16.5 | 451 | 451 | 319 |
| Myricetin 3-rhamnoside | Mrh | 16.9 | 465 | 465 | 319 |
| Quercetin 3-galactoside | Qgal | 17.1 | 465 | 465 | 303 |
| Quercetin 3-glucoside | Qglc | 17.5 | 465 | 465 | 303 |
| Quercetin 3-glucuronide | Qglr | 17.9 | 479 | 479 | 303 |
| Quercetin 3-arabinopyranoside | Qarap | 18.7 | 435 | 435 | 303 |
| Kaempferol 3-galactoside | Kgal | 18.8 | 449 | 449 | 287 |
| Quercetin 3-arabinofuranoside | Qaraf | 19.2 | 435 | 435 | 303 |
| Kaempferol-3-glucoside | Kglc | 19.6 | 287 | 449 | 287 |
| Quercetin 3-rhamnoside | Qrh | 19.7 | 303 | 449 | 303 |
| Kaempferol 3-glucuronide | Kglr | 20.1 | 463 | 463 | 287 |
| Kaempferol 3-arabinofuranoside | Karaf | 21.1 | 419 | 419 | 287 |
| Kaempferol 3-rhamnoside | Krh | 22.2 | 433 | 433 | 287 |
| **Epicuticular flavonoid aglycones** | | |  |  |  |
| Quercetin | F1 | 25.3 | 303 | 303 |  |
| Apigenin | F2 | 28.2 | 271 | 271 |  |
| Flavonoid aglycone 3 | F3 | 29.4 | 331 | 331 |  |
| Flavonoid aglycone 4 | F4 | 29.5 | 347 | 347 | 327 |
| Flavonoid aglycone 5 | F5 | 30.7 | 361 | 361 | 323 |
| Flavonoid aglycone 6 | F6 | 31.9 | 315 | 315 |  |
| Flavonoid aglycone 7 | F7 | 32.3 | 331 | 331 |  |
| Flavonoid aglycone 8 | F8 | 32.5 | 345 | 345 | 279, 329 |
| Flavonoid aglycone 9 | F9 | 32.7 | 361 | 361 |  |
| Flavonoid aglycone 10 | F10 | 33.8 | 375 | 375 |  |
| F11, Genkwanin | F11 | 33.9 | 285 | 285 |  |
| Flavonoid aglycone 12 | F12 | 34.6 | 315 | 315 |  |
| Flavonoid aglycone 13 | F13 | 35.1 | 331 | 331 |  |
| Flavonoid aglycone 14 | F14 | 36.2 | 345 | 345 |  |
| Flavonoid aglycone 15 | F15 | 45.1 | 357 | 357 |  |
| **Epicuticular triterpenoids** |  |  |  |  |  |
| Triterpenoid 1 | T1 | 43.5 | 527 | 527 | 423 |
| Triterpenoid 2 | T2 | 43.9 | 603 | 603 | 543, 638 |
| Triterpenoid 3 | T3 | 44.7 | 341 | 341 | 622, 569 |
| Triterpenoid 4 | T4 | 44.8 | 603 | 603 | 527, 423 |
| Triterpenoid 5 | T5 | 45.6 | 603 | 638 | 603 |
| T6, Papyriferic acid derivative | T6 | 49.7 | 545 | 545 | 477, 441, 627, 605 |
| T7, Papyriferic acid | T7 | 50.7 | 527 | 527 | 475, 441, 423, 627 |
| T8, 12-O-Acetyl-3-O-malonylbetulafolientriol | T8 | 54.8 | 571 | 571 | 511, 606, 588 |
|  |  |  |  |  |  |

Triterpenoids were annotated based on mass spectra. Papyriferic acid (T7) and its derivative (T8) had the indicative ion of ocotillol-type triterpenoids (143) in their spectra when run with a mass range of 50-1610 m/z. Epicuticular flavonoid aglycones include quercetin that can also be intracellular.

## Figures


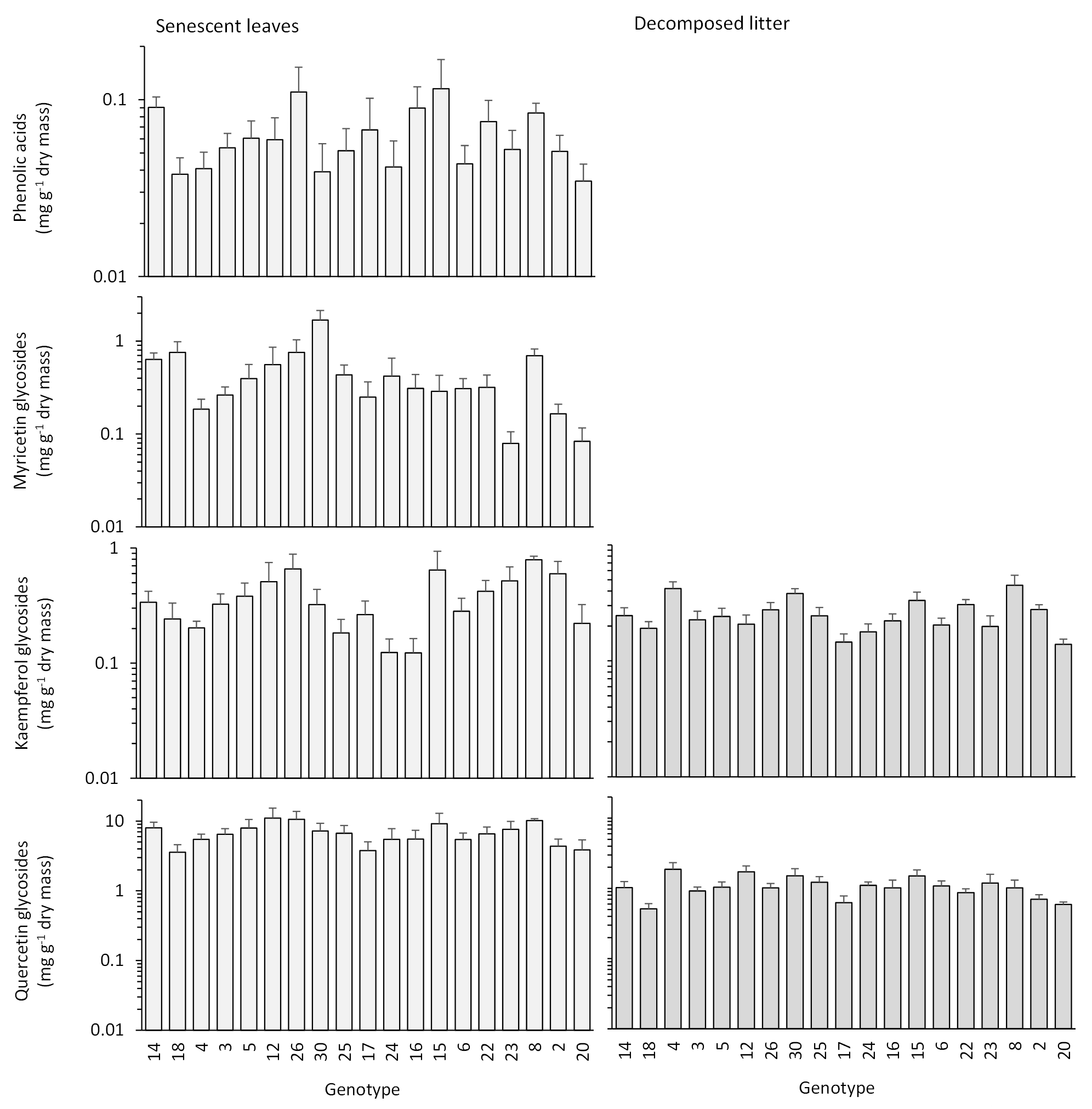


**Supplementary Figure 1.** Concentrations of intracellular phenolic subgroups: phenolic acids, myricetin glycosides, kaempferol glycosides and quercetin glycosides (mean+SE, n=5-6) in the senescent leaves and decomposed litter of 19 *Betula pendula* genotypes (arranged in a decreasing order of senescent leaf tannin concentrations in Fig. 2).

**Supplementary Figure 2.** Correlations between the mean concentrations of epicuticular triterpenoids, epicuticular flavonoid aglycones, condensed tannins and lignin in the senescent leaves of 19 *Betula pendula* genotypes.
